# Repetitive Negative Thinking Processes Account for Gender Differences in Depression and Anxiety During Adolescence

**DOI:** 10.1007/s41811-022-00133-1

**Published:** 2022-02-26

**Authors:** Fabiola Espinosa, Nuria Martin-Romero, Alvaro Sanchez-Lopez

**Affiliations:** 1 Department of Personality, Evaluation and Clinical Psychology, Faculty of Psychology, Complutense University of Madrid, Somosaguas Campus 28223, Madrid, Spain; 2 Department of Educational Sciences, University of Alcala, Alcalá de Henares, Spain

**Keywords:** Repetitive negative thinking, Depression, Anxiety, Adolescence, Gender differences

## Abstract

Rumination and worry are vulnerability factors involved in the early development of depression and anxiety during adolescence, particularly in girls. Current views conceptualize rumination and worry as transdiagnostic forms of repetitive negative thinking (RNT). However, most of research has analyzed them separately, without considering gender differences. We analyzed common and specific roles of rumination and worry in accounting for depressive and anxiety symptom levels overall and as a function of gender in adolescents (*N* = 159). Rumination and worry items were loaded into separate RNT factors. Girls showed a higher use of rumination and worry and higher levels of depression and anxiety than boys. Structural equation modeling supported that both RNT factors accounted for gender differences in symptom levels: rumination was the strongest mediator for depression and worry the strongest mediator for anxiety. Our findings support both general and specific contributions of RNT to account for affective symptomatology during adolescence, particularly in girls.

**Supplementary Information:**

The online version contains supplementary material available at 10.1007/s41811-022-00133-1.

Adolescence is a crucial developmental period, involving a profound amount of change in all individual domains: biological, cognitive, psychosocial, and emotional. Most of these changes (e.g., continued physical growth, behavioral and social development, sexual maturation) (Blakemore & Mills, 2014) occur simultaneously, making the transition from childhood to adulthood an emotionally intense developmental period and a unique window of vulnerability for the onset of emotional disorders (Rapee et al., 2019). Anxiety disorders, such as generalized anxiety, are the most prevalent disorders during adolescence, particularly among girls (Vallance & Fernandez, 2016). Further, mood disorders such as depression also have a substantial onset during adolescence, and, as with anxiety disorders, their prevalence is particularly high for girls (McLaughlin & King, 2015). Anxiety and depression generate both substantial emotional, social, and academic impacts in adolescents. Both forms of emotional disorders are associated with high risk for substance abuse (Gobbi et al., 2019) and future suicidal behavior in adulthood (Beesdo-Baum & Knappe, 2012), as well as high risk for additional forms of psychopathology during adolescence (Orgiles et al., 2019) and recurrence of emotional disorders during adulthood (Copeland et al., 2014; Essau et al., 2018). The high impact of depression and anxiety during adolescence and its continuity in adult life both highlight the need of understanding the mechanisms involved in their early onset during adolescence, particularly among girls.

## Repetitive Negative Thinking Processes: Specific or Common Predictors of Depression and Anxiety?

Repetitive negative thinking (RNT) is a common feature of both depression and anxiety. RNT can take the form of rumination (Nolen-Hoeksema et al., 2008) and/or excessive worry (Borkovec et al., 1983), two forms of unproductive RNT that have been typically related to depression and anxiety disorders, respectively. Rumination is defined as a form of RNT comprising a repetitively and passive focus on symptoms of discomfort or distress, as well as on the possible causes and consequences of these symptoms, not trying to solve or change the circumstances that surround them (Nolen-Hoeksema et al., 2008). Rumination impairs problem-solving, reduces motivation to initiate instrumental behaviors, and reduces social support (Nolen-Hoeksema et al., 2008; Lyubomirsky et al., 2015). Specifically brooding rumination, comprising passive and judgmental repetitive thoughts about one’s mood (Treynor et al., 2003), has been identified as a particularly maladaptive form of RNT, and it has been largely associated with the onset of depressive symptoms (Nolen-Hoeksema et al., 2008; Treynor et al. 2003; Schoofs et al., 2010). Whereas the conceptualization of rumination and the derived empirical research on its emotional impact have been mostly referred to their relationship with depression, worry has been typically considered as a key risk factor for the development of anxiety disorders. Excessive worry, as originally defined by Borkovec et al. (1983), comprises a chain of future-oriented repetitive thoughts and images, negatively affect-laden and relatively uncontrollable. These thoughts represent attempts to engage in mental problem-solving on future issues whose outcomes are uncertain and contain the possibility of one or more negative outcomes. Consequently, worry has been related to fear processes (Borkovec et al., 1983; see also Makovac et al., 2018), cognitive biases to threat (Hirsch et al., 2011), and, ultimately, to the onset of anxiety symptoms (Costello et al., 2005; Pine et al., 1998).

Despite the differences in terms of their content (past- vs. future-oriented), rumination and worry present multiple formal commonalities (i.e., repetitive nature, negative focus) that have led to consider them as potential forms of a unique higher RNT transdiagnostic factor (Harvey et al., 2004). From this view, the tendency to use different forms of repetitive negative thoughts (RNT), either rumination or worry or both, would represent a central mechanism causally involved in emotion dysregulation (Ehring & Watkins, 2008), and, therefore, a risk factor for the development of both depression and anxiety. From this perspective, different forms of RNT such as rumination and worry would not reflect specific mechanisms of depression and anxiety risk, respectively, but be part of a general factor of transdiagnostic RNT contributing to the development of both types of emotional problems (Spinhoven et al., 2018). In line with this proposal, empirical research supports that rumination is not only associated with the onset and maintenance of depression, as originally proposed, but also with higher anxiety symptom levels both in adolescents and adults (e.g., Armstead et al., 2019; McLaughlin & Nolen-Hoeksema, 2011). Similarly, several studies have found that worry is not only associated with higher anxiety, but also with higher depressive symptom levels (Olatunji et al., 2013; Topper et al., 2017). These findings suggest that common RNT processes might account for the high comorbidity rates among anxiety and depressive disorders that are initiated during adolescence and then continued across adulthood. Relatedly, recent longitudinal research supports that increases in both forms of RNT, worry and rumination, predicts increases in symptoms of both depression and anxiety in adolescents (Young & Dietrich, 2015). Thus, a precise study of both common and specific features of these forms of RNT in non-clinical samples of adolescents is essential to understand the paths of vulnerability for early onset of depression and anxiety during adolescence and their comorbidity. However, research on this issue is still considerably limited. Furthermore, the use of these integrative approaches seems crucial as a mean of clarification for the existence of gender differences that have been extensively observed in both depression and anxiety during adolescence.

## RNT Processes as Mechanisms of Gender Differences in Depression and Anxiety During Adolescence

Extensive research supports gender differences in the prevalence of both mood and anxiety disorders, such as generalized anxiety. Women significantly experience more often these problems compared to men across the lifespan, with this gender gap already emerging during adolescence (Kwong et al., 2019; Ohannessian et al., 2016). Research on the mechanisms accounting for emerging gender differences in depression and anxiety risk during adolescence has considered how specific forms of RNT (i.e., either rumination or worry) account for specific higher rates of psychopathology in girls (i.e., depression or anxiety levels, respectively). This approach seems crucial. Establishing whether girls already start engaging in higher uses of RNT forms than boys during adolescence and whether such RNT forms account for an earlier emergence of symptoms in girls may serve to define more precise markers of risk detection at different ages and genders and to inform more effective prevention programs for adolescents. As for rumination, extensive studies support the existence of higher levels of rumination in women, both in adolescents (Hilt et al., 2010; Gomez-Baya et al., 2017; Muris et al., 2004) and in adults (Nolen-Hoeksema & Jackson, 2001). Higher levels of rumination in adolescent girls have been consistently linked to their higher levels of depressive symptomatology compared to adolescent boys (Gomez-Baya et al., 2017; Rood et al., 2009). Thus, a greater tendency to ruminate in girls has been postulated as a possible explanation to gender differences found for depression symptoms, emerging during adolescence, and persisting across adulthood (Gomez-Baya et al., 2017; Nolen-Hoeksema et al., 1999; Ottaviani et al., 2016; Shors et al., 2017). Several studies have also shown that girls report higher levels of anxiety than boys during adolescence (Beesdo-Baum & Knappe, 2012; McCauley et al., 2017; McLaughlin & King, 2015; Stapinski et al., 2015) and that they are more likely than boys to be diagnosed with anxiety disorders (McCauley et al., 2017). Similarly, some studies show that girls report higher levels of worry than boys during adolescence (Muris et al., 2004; Sweeny et al., 2019), suggesting that this form of RNT might also be accounting for gender differences in anxiety problems during adolescence.

Thus, research has solely considered the role of rumination to account for gender differences in depression rates during adolescence, whereas research on early onset of anxiety symptoms during adolescence has uniquely considered the potential accounting role of excessive worry. To date, no research has integrated the study of both forms of RNT, rumination and worry, to account for both gender differences in depression and anxiety levels during adolescence. This step seems fundamental, given current evidence suggesting a general contributing role of RNT processes to account for general increased risk for emotional dysfunctions during adolescence (e.g., Young & Dietrich, 2015).

## The Present Study

The present study aimed to analyze the understudied common and specific contributions of different forms of RNT (i.e., rumination, worry) to account for gender differences observed in depression and generalized anxiety symptoms during adolescence. We evaluated different forms of RNT, both rumination and worry, as well as depression and generalized anxiety levels in a non-clinical sample of adolescents in order to (a) determine common and specific dimensions of RNT processes of rumination and worry and its general contribution to anxiety and depression in adolescence, in line with previous research (Muris et al., 2004) while also (b) determining gender differences that can emerge in such RNT processes and examining their role in accounting for gender differences in depression and anxiety levels in adolescents with ages ranging between 12 and 17 years. First, we expected that rumination and worry would show a significant degree of covariation (hypothesis 1). Beyond this basic assumption, the loading of both dimensions into a common higher-order RNT factor vs. independent loadings into specific forms of rumination and worry was tested via factor analyses. Based on those results, different indices (global, RNT; and/or specific, rumination, worry) were considered to analyze the main questions under study regarding common and specific contributions of RNT processes to account for gender differences in depression and generalized anxiety. As a previous step, we considered to common and/or specific role of RNT processes in accounting for individual differences in depression and anxiety among adolescents in general. In line with previous research on this issue (Muris et al., 2004), it was expected that higher levels in specific forms of rumination would be more strongly linked to higher levels of depression than generalized anxiety (hypothesis 2), whereas the other way around, higher levels in specific forms of worry would be more strongly linked to higher levels of generalized anxiety than depression (hypothesis 3). As for gender differences, it was expected that girls compared to boys would show higher levels of RNT factors (hypothesis 4), as well as higher levels of both depression and generalized anxiety levels (hypothesis 5). Relatedly, in order to fill the gap on research considering separate forms of RNT as specific mechanisms of gender differences in depressive and generalized anxiety symptoms, the role of empirically supported common and specific factors of RNT was modeled together into a structural equation model, considering them as potential mediators of the role of gender on differences in both anxiety and depressive symptom levels.

## Methods

### Participants and Procedure

One hundred fifty-nine adolescents (61 boys and 98 girls) were recruited from several high schools in Spain. The average age of the sample was 14.64 years (SD = 1.51, range 12–17 years).

High schools were first contacted and sent a cover letter with all the information about the research. High schools that agreed to participate were then contacted to review the protocol of data collection. Adolescents whose parents agreed that they could participate in the study received full instructions and the questionnaire package via email. First, adolescents were informed that their responses were anonymous and confidential and that participation was voluntary. All the adolescents consented to participate in the study. They then completed the questionnaires online, through a survey in Google Forms, supplied via a link in the contact email. Prior to starting the questionnaire administration, participants were informed of the importance of maintaining a quiet environment free from distractions to complete the questionnaires as well as of the importance of following the instructions for each questionnaire carefully. Before completing the questionnaires, gender was assessed asking adolescents to reply to the question: *Indicate the gender with which you feel most identified? (boy/girl)*. The questionnaires used were those ones explained in the next Measures section. No other measures were used.

Data collection was carried out in the period between November 2019 and January 2020, before the COVID-19 pandemic. The study was conducted in accordance with the Declaration of Helsinki. Consent forms were collected both from the parents in the first place and then from the adolescents before starting their participation. The project was reviewed by the evaluation committee of the Masters’ program of Clinical Psychology of Complutense University of Madrid.

### Measures

#### Rumination

The *Ruminative Responses Scale* (RRS; Nolen-Hoeksema & Morrow, 1991; Spanish adaptation: Hervas, 2008) is a questionnaire that assesses the presence of a ruminative response style, as a pattern of RNT comprising a focus on the causes and consequences of negative mood. This assessment tool is largely considered a significant predictor of depressive symptoms (Hervas, 2008; Nolen-Hoeksema & Morrow, 1991). It consists of 22 items, coded using a Likert scale of 4 points, and its scores range between 22 and 88. The RRS total score was used to obtain a global index of rumination. The scale also allowed to index two separate subfacets of rumination, reflection and brooding. In the present study, we were interested in the use of the brooding subscale to test hypotheses regarding common and specific forms of RNT and their contributions to emotional symptom levels. The Spanish version of the RRS has shown previous good reliability both in terms of internal consistencies (global rumination scale, *α* = .93; subscale of *brooding*, α = .80; subscale of reflection, α = .74), as well as in terms of test–retest reliability (5 months, *r* = .56; 1 year, *r* = .54) (Hervas, 2008). Internal consistencies were very good in the present study and are reported in Table 1, as well as for the rest of questionnaires.

**Table 1:** Means, standard deviations (in brackets), and internal consistencies of the variables in the study

	Total (*N* = 159)	Girls (*N* = 98)	Boys (*N* = 61)	α
Rumination (RRS total; range, 22 to 88)	47.58 (13.76)	49.73 (14.24) *	44.13 (12.29)	0.92
Brooding (RRS subscale; range, 5 to 20)	11.32 (3.62)	11.70 (3.91) +	10.70 (3.01)	0.72
Worry (PSWQ; range, 16 to 80)	47.80 (12.52)	50.45 (12.62) **	43.54 (11.19)	0.87
Depression (CES-D; range, 8 to 32)	8.37 (5.63)	9.43 (5.88) **	6.65 (4.78)	0.88
Anxiety (GAD; range, 0 to 21)	8.45 (5.24)	9.37 (5.38) **	6.98 (4.70)	0.86

Statistical significance of differences between girls and boys. +*p* < .10; *p < .05; **p < .01

#### Worry

The *Penn State Worry Questionnaire* (PSWQ; Meyer et al., 1990; Spanish adaptation: Sandin et al., 2009) is a self-report questionnaire that assesses the general tendency to worry. It comprises 16 items, coded using a Likert scale from 1 to 5, with total scores ranging from 16 to 80. Psychometric properties of the Spanish version, used in this study, are good, showing a high value of Cronbach’s alpha (*α* = .90) and an appropriate 1-month test–retest stability (r = .82; Sandin et al., 2009). The internal consistency in the current study was good (see Table 1).

#### Depressive Symptoms

The Center for Epidemiologic Studies Depression Scale (CES-D; Radloff, 1977; Spanish adaptation: Vázquez et al., 2007) is a self-report measure of depression symptom levels, referred to the last week. The Spanish version in the 8-item version was used in this study (Vázquez et al., 2007), which has proven its utility to detect depressive symptomatology and risk factors among non-clinical populations, at various ages. It comprises 8 items (CES-D 8), completed using a Likert scale of 4 options and with scores ranging between 8 and 32. The version used in this study has shown good psychometric properties, with a Cronbach’s alpha coefficient ranging between 0.80 and 0.92 in different populations and sociocultural contexts (e.g., Torres-Lagunas et al., 2015; Van de Velde et al., 2009), and it is widely used in studies testing the prevalence of depression in the general population for various age and gender groups. The internal consistency in the current study was good (Table 1)

#### Anxiety Symptoms

The Generalized Anxiety Disorder scale (GAD-7) (Spitzer et al., 2006; Spanish adaptation: Garcia-Campayo et al., 2010) is a self-report measure that assesses the severity of anxiety symptom levels during the last 2 weeks. It consists of 7 items, coded in a Likert scale of 4 options. Total scores range from 0 to 21. The psychometric properties in the Spanish version are very good (Garcia-Campayo et al., 2010), showing an excellent internal consistency (*α* = 0.94), and good test–retest stability 1 week later (*r* = 0.84). The GAD-7 has also proven to be a reliable and valid instrument for the estimation of individual differences in general anxiety levels. In this regard, the convergent validity with other general anxiety instruments such as, for example, the subscale of anxiety in the Depression, Anxiety and Stress Scale 21 (DASS) (Gloster et al., 2008) is significantly high (*r* = 0.77; Kertz et al., 2013). In the present study, internal consistency of the instrument was good (see Table 1).

### Data Analysis Plan

To test hypothesis 1, analyses of bivariate correlations as well as a series of factor analyses were conducted to estimate the degree of covariance between rumination and worry, as well as to test the support for a general loading factor of RNT items into a single higher-order RNT factor vs. into specific factors of rumination and worry. In order to test hypotheses 2 and 3, correlations between rumination and worry and levels of depressive and anxiety symptomatology were computed, followed by tests for comparing the resulting correlation coefficients (Meng et al., 1992). In order to contrast hypotheses 4 and 5, *t* tests were performed comparing girls and boys in each of the variables. This was followed by an analysis of covariance including the variables of RNT as covariables in the tests of gender differences (independent variable) for each symptomatology variable (dependent variables).

**Fig. 1:**
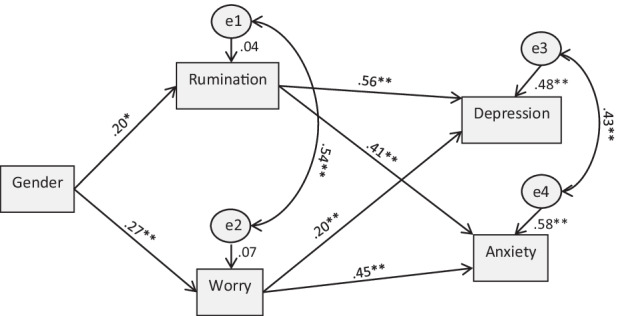
Model of the relationship among gender, RNT dimensions, and symptom outcomes. *Note.* Significant SEM model paths: *p < .05; **p < .01

Finally, and supported by those preliminary analysis, we tested the role of RNT factors in accounting for gender differences found in anxiety and depression symptom levels. To this aim, we constructed a structural equation model (SEM) to test supported RNT factors as intervening factors among gender and psychological symptom outcomes. SEM accounts for multiple accumulative relationships among variables, while handling numerous sources of variance, and allows to test the directional hypothesized relationships (i.e., gender ➔ RNT ➔ symptoms). RNT and symptom variables in the model comprised total scale scores. Maximum-likelihood estimation (ML) was used to estimate the model. Model fit was assessed using the chi-square goodness of fit statistic, the root mean-square error of approximation (RMSEA) (Browne & Cudeck, 1992), the normed fit index (NFI) (Bentler and Bonnet, 1980), and the comparative fit index (CFI) (Bentler, 1990). Model fit criteria were considered according to the ones established in previous research (CFI ≥ .95 and RMSEA ≤ .06 (Hu & Bentler, 1999); NFI ≥ .9 (Marsh & Hau, 1996)). The model included gender as the exogenous predicting variable. The endogenous (i.e., dependent) variables in the model were the self-reported symptoms of depression and anxiety. The constructs of RNT dimensions, rumination and worry, were considered as exogenous variables (predictors of depression and anxiety) and as endogenous variables predicted by gender (see Fig. 1). Given the observed covariation among RNT dimensions, as well as among depression and anxiety levels, error terms among each set of variables were allowed to correlate in the model. Indirect effect paths (i.e., gender ➔ RNT ➔ symptoms) within the model were tested using 2000 bootstrap samples and 95 bias-corrected confidence intervals. All the analyses were performed in SPSS. The Analysis of Moment Structures (AMOS) module (Version 25.0; Arbuckle, 2017) of the SPSS statistical package was used to complete the analyses concerning the final SEM.

## Results

### Preliminary Analyses

Descriptives and internal consistencies for each variable are reported in Table 1, for the total sample as well as for each separate gender group. We first examined skewness and kurtosis for each variable to confirm normality of data distributions. Indices were below ± 2 in all cases, supporting normal distributions (Schumacker & Lomax, 2012). Further, bivariate correlations, presented below, were not indicative of any correlation > .9, indicating no multicollinearity issues among the variables under study. As mentioned above, all questionnaires were reliable in terms of the internal consistencies of the factors, obtaining Cronbach’s alpha coefficients ranging between 0.72 and 0.92.

### Correlations Between Rumination and Worry

As for the first hypothesis (i.e., the relation between rumination and worry will be significant degree of covariation), the results confirmed a significant positive correlation both between worry and the rumination total score, *r =* .56 and *p <* .001, as well as between worry and the subcomponent of ruminative *brooding*, *r =* .51 and *p <* .001. Thus, analyses indicated a large degree of covariance between the dimensions of RNT under study, although not a clear overlap between the two constructs of RNT.

### Factor Analysis

In order to further test common vs specific loadings of rumination and worry forms of RNT, and according to sample size in the study, an exploratory factor analysis was chosen, extracting dimensions within a pre-defined subgroup of items of each RNT scale. Regarding the rumination scale, specifically, items assessing the maladaptive subcomponent of ruminative brooding were selected, given its stronger relation to the onset and maintenance of emotional symptomatology in the previous research (see Nolen-Hoeksema et al., 2008). Regarding the worry scale, preliminary analysis determined that the reversed items in this scale were loaded into a separate specific factor, without any loading into the rest of worry components derived from the rest of items of the scale, thus reflecting an artifact derived from the inverse formulation of these items.

The final factor analysis of the main components was therefore performed (Oblimin rotation, as correlated factors were hypothesized, in line with previous research on this issue: Muris et al. (2004)), selecting the items that formed the brooding factor of the RRS scale and the non-reversed items of the PSWQ scale. Two factors were found with eigenvalues greater than 1.00 (6.58 and 3.92), with the scree test pointing to a two-factor solution, which explained 53% of the variance. As can be seen in Table 2, all non-inverted items from the PSWQ scale were loaded consistently on the first factor, whereas all the elements of the brooding RRS subscale were loaded on the second factor. Based on these findings, not supporting a common loading into a higher global RNT factor, subsequent analyses were performed considering rumination and worry factors separately while controlling for their covariance.

**Table 2:** Rotated factor loadings obtained with exploratory factor analysis (Oblimin rotation) of the non-inverted items of PSWQ and those referred to the brooding subcomponent in the RRS. In bold, items loading into each factor

	Factor 1	Factor 2
PSWQ13. I notice that I have been worrying about things	**.840**	−.133
PSWQ14. Once I start worrying, I cannot stop	**.787**	.032
PSWQ7. I am always worrying about something	**.739**	.103
PSWQ5. I know I should not worry about things, but I just cannot help it	**.731**	.085
PSWQ15. I worry all the time	**.708**	.144
PSWQ6. When I am under pressure, I worry a lot	**.720**	−.023
PSWQ4. Many situations make me worry	**.692**	.080
PSWQ9. As soon as I finish one task, I start to worry about everything else I have to do	**.745**	−.157
PSWQ12. I have been a worrier all my life	**.655**	.171
PSWQ16. I worry about projects until they are all done	**.687**	−.062
PSWQ2. My worries overwhelm me	**.614**	.183
RRS15. Think “Why do I have problems other people don’t have?”	−.070	**.794**
RRS16. Think “Why can’t I handle things better?”	.085	**.737**
RRS13. Think about a recent situation, wishing it had gone better	−.045	**.664**
RRS10. Think “Why do I always react this way?”	.059	**.576**
RRS5. Think “What am I doing to deserve this?”	.111	**.547**

*N* = 159. Items selected from the questionnaires Ruminative Responses Scale, brooding subscale (RRS), and Penn State Worry Questionnaire, non-reversed items (PSWQ)

### Strength of Correlations Between RNT Processes and Anxiety and Depression

Analysis of bivariate correlation showed significant positive correlations between the rumination total score and both depression, *r =* .67 and *p <* .001, and anxiety symptom levels, *r =* .66 and *p <* .001. Similarly, worry was significantly positive correlated with both depression, *r =* .51 and *p <* .001, and anxiety symptom levels, *r =* .68 and *p <* .001.

To contrast the hypotheses 2 (i.e., rumination will be more strongly linked to higher levels of depression than anxiety) and 3 (i.e., worry will be more strongly linked to higher levels of anxiety than depression), tests for comparing correlation coefficients were performed (see Meng et al., 1992; also Muris et al., 2004). Hypothesis 2 was not supported, finding an equal magnitude of correlation of the rumination total score with depression and with anxiety levels, *z =* −0.11 and *p =* .91. In contrast, hypotheses 3 was supported, with worry being significantly more related to anxiety than to depression levels, *z =* 2.35 and *p =* .02.

### Gender Differences

To test hypothesis 4 (i.e., girls will show higher levels than boys in both RNT dimensions) and 5 (i.e., girls will show higher levels than boys in both depressive and anxiety symptomatology), *t* tests were conducted to compare boys and girls in each of the variables. The mean differences between boys and girls in depression (*t*(157) *=* −3.11; *p =*.0 02; *d* = .51) and anxiety (*t*(157) *=* −2.86; *p =* .005; *d* = .47), as well as in the rumination total score (*t*(157) *=* −2.54; *p* = .012; *d* = .41) and worry (*t*(157) *=* −3.51; *p =* .001; *d* = .57), were all statistically significant.

Provided that gender differences were supported not only in terms of symptom level measures but also in the two RNT dimensions, a series of steps were followed to test the intervening role of RNT in accounting for gender differences in symptomatology in adolescents. A preliminary step comprised analyses of covariance to test whether RNT dimensions accounted for the observed variance between genders in each symptomatology outcome. Thus, gender was introduced as the independent variable and the two dimensions of RNT — rumination total score and worry — as covariates in two separate models for depression and anxiety levels as outcomes. Results indicated that gender differences previously found in depression and anxiety outcomes in the *t* tests did no longer reach significance after that RNT variables were controlled as covariates in the corresponding model, depression (*F*(1,155) = 1.91, *p =* .17, *η*_*p*_^*2*^* =* .01) and anxiety (*F*(1,155) = 0.16, *p =* .69, *η*_*p*_^*2*^* =* .01). Thus, it might be considered that the differences found between boys and girls in symptomatology outcomes could be explained by observed gender differences in both RNT dimensions. This full model was conversely tested in the final structural equation model.

### RNT Processes Accounting for Gender Differences in Depression and Anxiety: Structural Equation Modeling

The results of the SEM model with path coefficients (i.e., regression standardized weights) are shown in Fig. 1. The fit of the model was excellent, Chi-square goodness of fit (degrees of freedom, 2) = 1.99, *p =* .37; RMSEA = .01; NFI = .98; CFI = .99.

Gender was predictive of both differences in the rumination total score (*β =* .20 *p <* .05) and worry (*β =* .27, *p <* .001), whereas the RNT dimensions were both significantly predictive of depression (rumination, *β =* .56, *p <* .001; worry, *β =* .20, *p <* .01) and anxiety levels (rumination, *β =* .41, *p <* .001; worry, *β =* .45, *p <* .001). In terms of indirect effects within the model (i.e., gender → RNT → symptom level), results are summarized in Table 3. The four indirect effect models were statistically significant, supporting the prediction that gender differences in RNT processes ultimately accounted for gender differences in both depression and anxiety outcome levels. Within that general common contribution, analyses showed that rumination had a more substantial mediating effect in accounting for the indirect effect of gender on depression levels, whereas worry had a more substantial mediating effect in accounting for the indirect effect of gender on anxiety levels.

**Table 3:** Analysis of the indirect effects within the SEM explanatory model

Indirect effect parameter	Estimate	Lower confidence interval	Higher confidence interval	*p*
Gender ➔ rumination ➔ depression	1.287	.332	2.338	.009
Gender ➔ rumination ➔ anxiety	.878	.202	1.648	.010
Gender ➔ worry ➔ depression	.608	.208	1.257	.001
Gender ➔ worry ➔ anxiety	1.303	.611	2.218	.001

## Discussion

The present study examined the role of different forms of RNT, rumination and worry, in accounting for gender differences in depressive and anxiety symptom levels in a non-clinical sample of adolescents. Analyses with the full sample indicated a high level of covariation between both dimensions of RNT. In line with previous studies (e.g., Muris et al., 2004), our results supported a high degree of co-occurrence among rumination and worry in adolescence, although not pointing out to a common general loading into a global RNT factor. This possibility was further contrasted by exploratory factor analyses that supported a two-factor solution explaining 53% of variance in RNT items, with each of these factors representing specific forms of worry or rumination. Therefore, in relation to hypothesis 1, findings support the consideration of rumination and worry as specific dimensions of RNT, although their high covariance indicates the need of considering both common and specific contributions of these RNT forms to psychological functioning (i.e., depression and anxiety levels) in adolescents. Future research in this issue may thus benefit of using bifactor approaches (see Taylor & Snyder, 2021), in order to consider both the contributions of the specific RNT dimensions and the contribution of their common variance in the contribution to gender differences in the development and maintenance of psychopathology during adolescence.

In relation to the former claim (i.e., hypotheses 2 and 3), whereas analyses showed positive significant correlations of both RNT forms (rumination, worry) with both depressive and anxiety symptom levels, symptom specificity was supported for worry but not for rumination, in the general sample. Thus, rumination had similar a strength of associations with both depression and anxiety levels, pointing to a common role in contributing to both forms of emotional psychopathology, in general. In contrast, the contribution of worry was significantly higher for anxiety symptom than for depressive symptom levels. These results reveal the importance of worry for early anxiety onset. As for rumination, common contributions to depression and anxiety are in line with former studies supporting it as a predictive factor of both depressive and anxiety symptoms (McLaughlin & Nolen-Hoeksema, 2011). In particular, in the meta-analysis of Özlem Schäfer et al. (2017), and in line with the results obtained in the present study, it was noted that there was a similar contribution of rumination in the prediction of both depression and anxiety symptom levels.

From these general effects, it is crucial to understand gender differences in RNT processes (hypothesis 4) and how they account for observed differences between girls and boys in terms of their levels of depression and anxiety experienced during adolescence (hypothesis 5). In line with previous research in these issues (Gomez-Baya et al, 2017; Hilt et al., 2010; McCauley et al., 2017; McLaughlin & King, 2015), our results showed gender differences in both RNT dimensions and symptoms, with girls engaging more in rumination and worry and experiencing higher depression and anxiety levels than boys. Importantly, after controlling for RNT dimensions, gender was no longer a predictor of individual differences symptomatology levels, suggesting the possibility that gender differences in adolescent symptomatology could be accounted by gender differences in RNT processes. A full SEM was constructed, modeling these pathways and testing the indirect effects of gender in each symptom outcome via each form of RNT (i.e., gender ➔ RNT ➔ symptoms). Intervening pathways of RNT dimensions were supported, with both rumination and worry measures accounting for gender differences in both depression and anxiety levels. Interestingly, beyond such common RNT effects to account for differences between girls and boys in symptom levels, analyses also pointed out specificity effects (i.e., higher indirect effect estimates) of rumination and worry in accounting for gender differences in depression and anxiety, respectively. Such integrative model had not been previously considered in research on RNT in adolescence, and the resulting evidence may have relevant implications.

Our results highlight that both dimensions of RNT can be particularly relevant and must be considered together to gain a better knowledge on their common and specific contributions to early onset of anxiety and depressive symptoms during adolescence, in general, and particularly in girls. Whereas our evidence concerns cross-sectional pathways, the clear fit of the structural equation model and the support for the claim that RNT processes exert both common and specific contributions to account for gender differences in emotional symptomatology warrants future longitudinal designs to test such effects prospectively. The results of this study are in line with the model of Nolen-Hoeksema et al. (1999), which proposes that the greater tendency of girls to ruminate may underlie the observed gender differences in depressive symptoms. Evidence also suggests that the occurrence of greater challenges in early adolescence for girls, combined with girls’ greater tendency to focus on their emotions, may increase their likelihood of occurrence of emotional disorders (Hilt et al., 2010; Lyubomirsky et al., 2015). Previous research has shown that rumination starts to interact with the occurrence of stressful life events to predict emotional psychopathology around 12 years of age (Felton et al., 2013). The transition from early to middle adolescence is a critical period, and, in the same way, changes in ruminative responses to stress have been observed (Felton et al., 2019), which typically precedes depression onset in later ages (Merikangas et al., 2009). Likewise, worry starts to be a common phenomenon in non-clinical populations from 8 to 13 years (Muris et al., 1998), also preceding the occurrence of anxiety disorders (Lijster et al., 2016). Whereas such temporal pathways among RNT dimensions and symptoms occurrence have been typically considered separately, our model support a further integrative longitudinal approach that may inform the co-occurrence of depression and anxiety increases across adolescence. This may help to disentangle the differential effects of RNT dimensions as a function of gender. Specifically, multi-year follow-ups of RNT and symptom variables should be integrated into structural modeling to study how gender affects the growth of RNT processes on the development of subsequent symptom outcomes across time (for similar methodological approaches of latent growth modeling to mediation analysis, see, for instance, Cheong et al., 2003). Further extensive models should also consider the inclusion of the other well-established vulnerability factors in the study of the indirect pathways of impact of gender on symptoms’ development across adolescence. Such models should integrate the study of cognitive factors such as dysfunctional attitudes and negative processing biases, as they have both reported to further account for gender differences in affective symptoms in adolescents (see, for instance, Meiser & Esser, 2017; Woody et al., 2019).

Findings from the current study and further evidence from future informed longitudinal research can clearly contribute to improve the specificity of assessment and treatment of emotional disorders in adolescents. One of the most promising approaches in this vein is the *rumination-focused cognitive behavioral therapy*, which includes functional analysis, experiential and imagery exercises, and behavioral experiments to change rumination into more adaptive thinking styles (Watkins et al., 2011). Initial studies support the effectiveness of these types of treatment (Hvenegaard et al., 2020). This intervention has been shown to reduce rumination levels in adolescents with major depressive disorder, leading to clinical improvement in depressive symptoms and promoting long-lasting improvements in the transition to adulthood (Jacobs et al., 2016). Our findings suggest that this type of intervention might benefit from integrating further modules for worry intervention and from considering gender differences to personalize interventions targeting one or both RNT factors. In this vein, further preventive interventions are being designed with the aim of reducing the probability of early anxiety and depression disorder onset by targeting different forms of RNT. In the study of Topper et al. (2017), the effectiveness of a preventive intervention for anxiety and depression disorders in adolescents and young adults (15–22 years old) was assessed, specifically focusing on excessive levels of RNT processes. These authors found that the preventive intervention reduced both worry and rumination levels and that such decreases mediated the reduction of anxiety and depressive symptomatology. These results reveal the importance of treatments focused on transdiagnostic risk factors of RNT to reduce the prevalence of both depressive and anxiety disorders. These approaches can benefit from the reported evidence for gender-based common and specific contributions of rumination and worry to different forms of emotional symptomatology across adolescence in the present study. This may help to personalize intervention modules and thus maximize their effectiveness.

The present study has some limitations. The sample did not have an equal distribution of ages in order to perform more specific analysis by age groups. Further studies, particularly using longitudinal approaches as the ones proposed above, may be highly informative to disentangle how supported overall paths manifest across the adolescence span. This ultimately will help to identify target periods for early RNT detection and symptom prevention and early treatment. Further, as noted above, our study was cross-sectional in nature. Although directional paths were supported from statistically solid structural equation modeling, further longitudinal studies informed by our current findings are necessary to replicate and extend current evidence. As for our study protocol, we were interested on the assessment of main RNT and symptom variables. It would be interesting that future research considered other psychosocial variables such as family structure, socioeconomic data, or previous psychopathology history, as possible moderators of the main observed effects, overall and specifically at different ages in the adolescence span. As for the instruments, the scales to assess rumination (RSS) and worry (PSWQ) are by definition closely linked to depressive and anxiety symptomatology, respectively. Thus, it could be argued that specificity findings might be dependent on the nature of the instruments used to index different sets of RNT processes (Spinhoven et al., 2015). Further research can benefit from contrasting common and specific loadings and contributions to early risk for psychopathology by integrating these measures as well as more general instruments of RNT such as the Repetitive Thinking Questionnaire (RTQ) (Mahoney et al., 2012). Finally, it is also important to take into account that we used a binary gender category in our study, where the constructs female and male were considered. This was done following previous research studying gender differences in rumination and worry and their relationships with depression and anxiety (e.g., Kwong et al., 2019; Ohannessian et al., 2016). However, future research should also address the study of non-binary gender categories, where adolescents with a non-binary gender identity, who do not identify with a binary classification, are considered

Despite these limitations, the present study provides empirical support for the role of RNT dimensions of worry and rumination as potentially mediating factors accounting for gender differences in depression and anxiety symptoms during adolescence. Apart from providing results in line with previous studies considering the role of RNT during adolescence in general (e.g., Muris et al., 2004; Spinhoven et al., 2018), the present study adds a gender perspective and a mechanistic model with clear implications for further advancement in the prevention of emotional symptomatology onset in adolescents, particularly in girls.

## Electronic supplementary material

Below is the link to the electronic supplementary material.

Supplementary file1 (DOCX 1040 KB)

Supplementary file2 (SAV 25 KB)
