# Supplementary material for: Repetitive Negative Thinking Processes Account for Gender Differences in Depression and Anxiety During Adolescence
Source: Int J Cogn Ther. 2022 Feb 26;15(2):115–33. doi: 10.1007/s41811-022-00133-1 (PMC8881790; doi:10.1007/s41811-022-00133-1)

**SPSS Syntax:**

DATASET ACTIVATE ConjuntoDatos1.

FREQUENCIES VARIABLES=Género Edad TGAD TCESD TPSWQ Rumia

/STATISTICS=STDDEV MEAN

/ORDER=ANALYSIS.

USE ALL.

COMPUTE filter_$=(Género = 1).

VARIABLE LABELS filter_$ 'Género = 1 (FILTER)'.

VALUE LABELS filter_$ 0 'Not Selected' 1 'Selected'.

FORMATS filter_$ (f1.0).

FILTER BY filter_$.

EXECUTE.

FREQUENCIES VARIABLES=Género Edad TGAD TCESD TPSWQ Rumia

/STATISTICS=STDDEV MEAN

/ORDER=ANALYSIS.

USE ALL.

COMPUTE filter_$=(Género = 2).

VARIABLE LABELS filter_$ 'Género = 2 (FILTER)'.

VALUE LABELS filter_$ 0 'Not Selected' 1 'Selected'.

FORMATS filter_$ (f1.0).

FILTER BY filter_$.

EXECUTE.

FREQUENCIES VARIABLES=Género Edad TGAD TCESD TPSWQ Rumia

/STATISTICS=STDDEV MEAN

/ORDER=ANALYSIS.

FILTER OFF.

USE ALL.

EXECUTE.

RELIABILITY

/VARIABLES=RRSitem1 RRSitem2 RRSitem3 RRSitem4 RRSitem5 RRSitem6 RRSitem7 RRSitem8 RRSitem9

RRSitem10 RRSitem11 RRSitem12 RRSitem13 RRSitem14 RRSitem15 RRSitem16 RRSitem17 RRSitem18 RRSitem19

RRSitem20 RRSitem21 RRSitem22

/SCALE('alfa RRS') ALL

/MODEL=ALPHA.

RELIABILITY

/VARIABLES=Item1PSWQinver PSWQitem2 Item3PSWQinver PSWQitem4 PSWQitem5 PSWQitem6 PSWQitem7

Item8PSWQinver PSWQitem9 Item10PSWQinver Item11PSWQinver PSWQitem12 PSWQitem13 PSWQitem14

PSWQitem15 PSWQitem16

/SCALE('alfa PSWQ') ALL

/MODEL=ALPHA.

RELIABILITY

/VARIABLES=CESDitem1 CESDitem2 CESDitem3 Item4Cesdinver CESDitem5 Item6Cesdinver CESDitem7

CESDitem8

/SCALE('alfa CESD') ALL

/MODEL=ALPHA.

RELIABILITY

/VARIABLES=GADitem1 GADitem2 GADitem3 GADitem4 GADitem5 GADitem6 GADitem7

/SCALE('alfa GAD') ALL

/MODEL=ALPHA.

CORRELATIONS

/VARIABLES=TPSWQ Rumia Brooding

/PRINT=TWOTAIL NOSIG

/MISSING=PAIRWISE.

CORRELATIONS

/VARIABLES=TGAD TCESD TPSWQ Rumia Brooding

/PRINT=TWOTAIL NOSIG

/MISSING=PAIRWISE.

FACTOR

/VARIABLES PSWQitem2 PSWQitem4 PSWQitem5 PSWQitem6 PSWQitem7 PSWQitem9 PSWQitem12 PSWQitem13

PSWQitem14 PSWQitem15 PSWQitem16 RRSitem5 RRSitem10 RRSitem13 RRSitem15 RRSitem16

/MISSING LISTWISE

/ANALYSIS PSWQitem2 PSWQitem4 PSWQitem5 PSWQitem6 PSWQitem7 PSWQitem9 PSWQitem12 PSWQitem13

PSWQitem14 PSWQitem15 PSWQitem16 RRSitem5 RRSitem10 RRSitem13 RRSitem15 RRSitem16

/PRINT INITIAL EXTRACTION ROTATION

/CRITERIA MINEIGEN(1) ITERATE(25)

/EXTRACTION PC

/CRITERIA ITERATE(25) DELTA(0)

/ROTATION OBLIMIN

/METHOD=CORRELATION.

T-TEST GROUPS=Género(1 2)

/MISSING=ANALYSIS

/VARIABLES=TGAD TCESD TPSWQ Rumia Brooding

/CRITERIA=CI(.95).

To calculate significance of the differences between two correlations coefficients we used: http://vassarstats.net/rdiff.html

Using the Fisher r-to-z transformation, this page calculated a value of z that can be applied to assess the significance of the difference between two correlation coefficients, ra and rb, found in two independent samples. To perform the calculation, we enter the respective values of r and n for the two samples into the designated cells, then we clicked the «Calculate» button.

Rumination – Depression and Anxiety:

Ra = .665

Rb = .672

Sample a = 159

Sample b = 159

Brooding – Depression and Anxiety:

Ra = .587

Rb = .563

Sample a = 159

Sample b = 159

Worry – Depression and Anxiety:

Ra = .681

Rb = .512

Sample a = 159

Sample b = 159

To perform the structural equation modeling (SEM) we used SPSS AMOS. Results are shown below:

Estimates (Group number 1 - Default model)

Scalar Estimates (Group number 1 - Default model)

Maximum Likelihood Estimates

Regression Weights: (Group number 1 - Default model)

Standardized Regression Weights: (Group number 1 - Default model)

Means: (Group number 1 - Default model)

Intercepts: (Group number 1 - Default model)

Covariances: (Group number 1 - Default model)

Correlations: (Group number 1 - Default model)


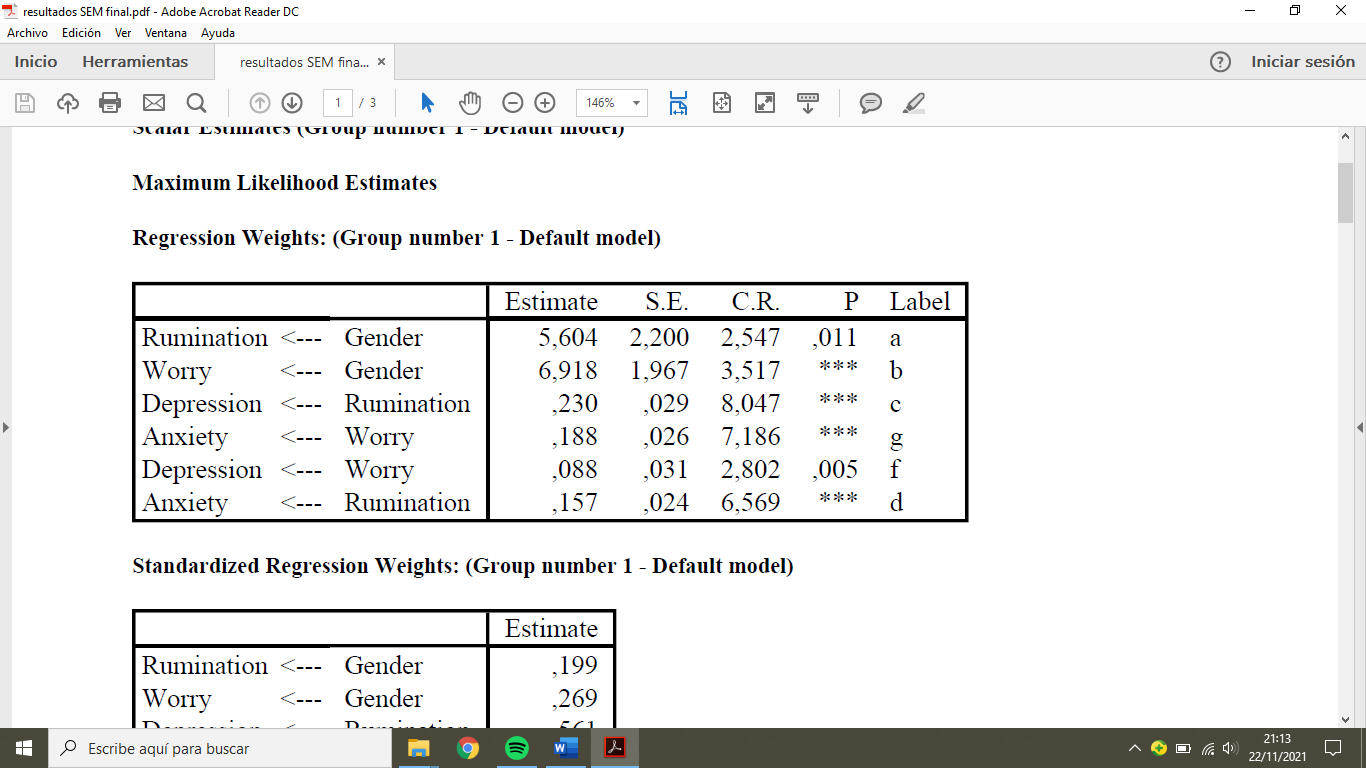


Standardized Regression Weights: (Group number 1 - Default model)


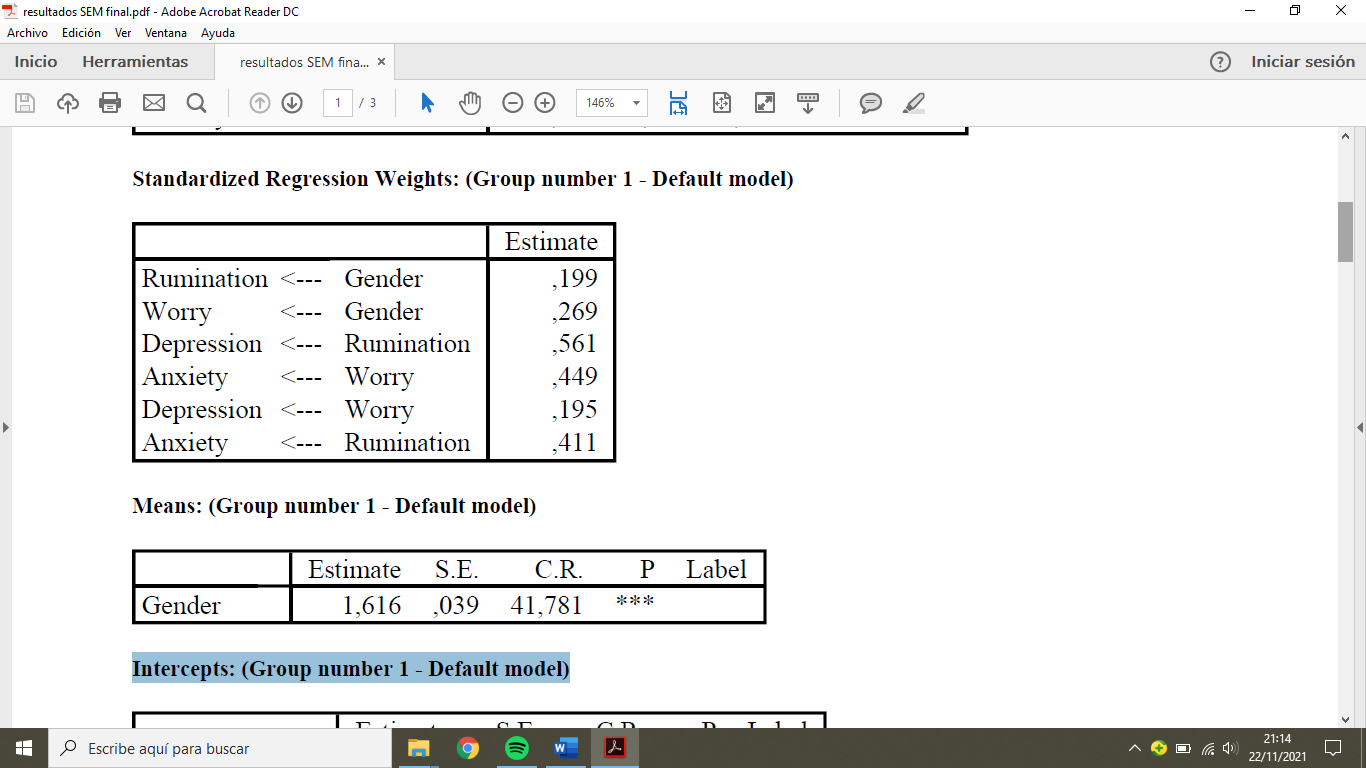


Means: (Group number 1 - Default model)


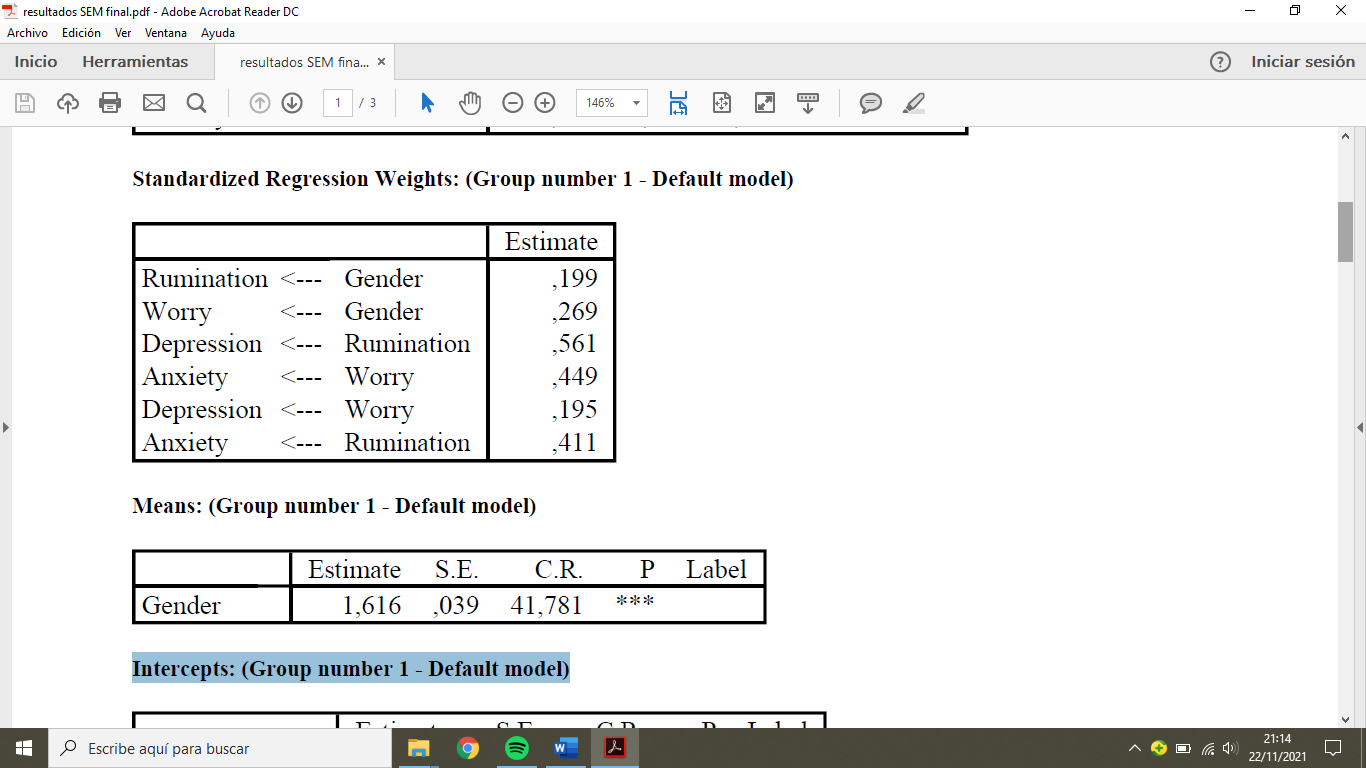


Intercepts: (Group number 1 - Default model)


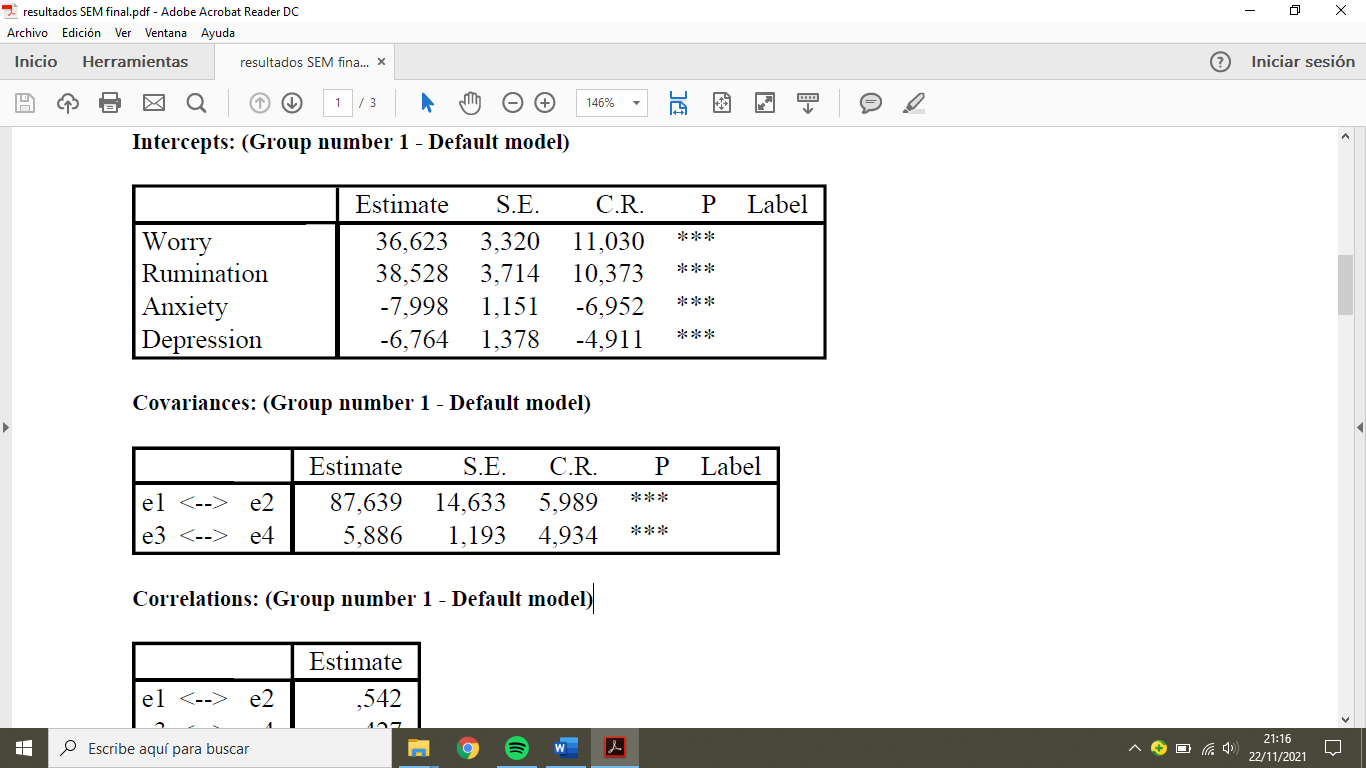


Covariances: (Group number 1 - Default model)


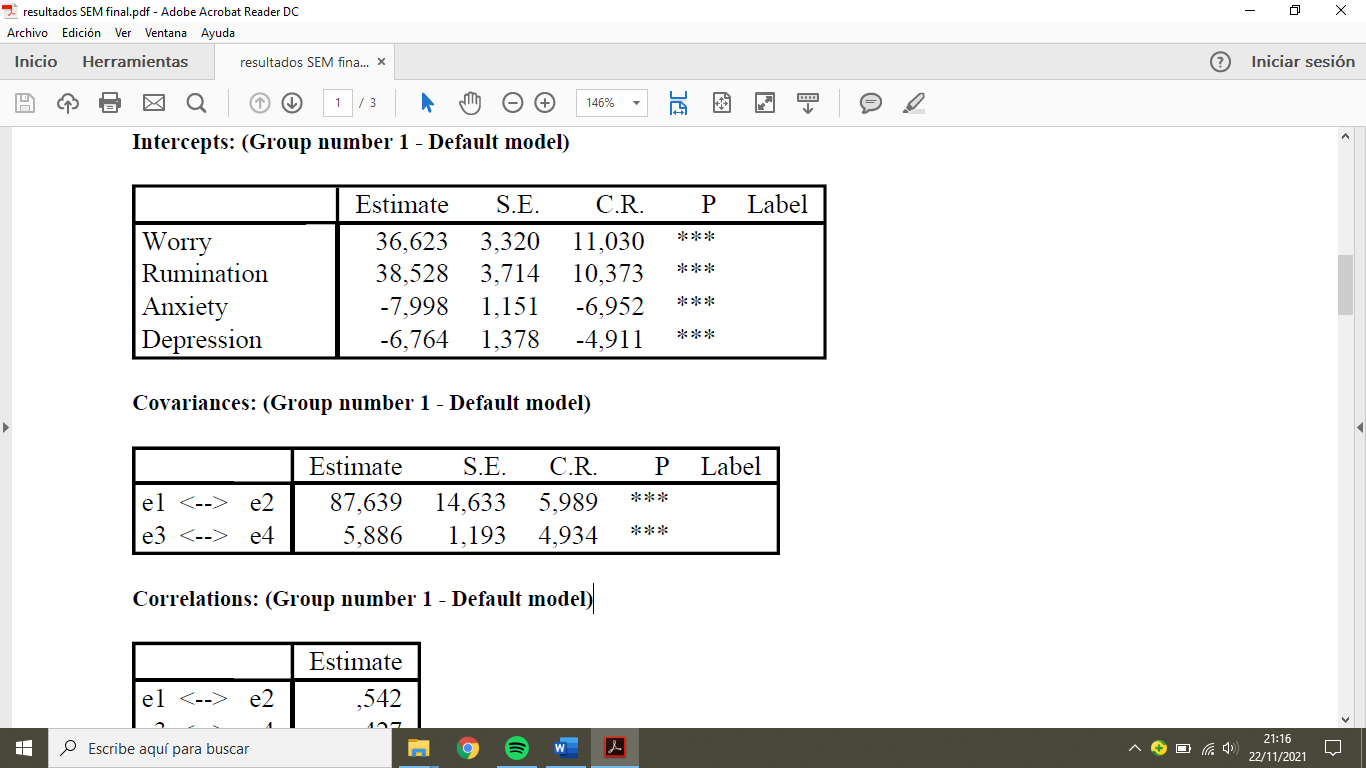


Correlations: (Group number 1 - Default model)


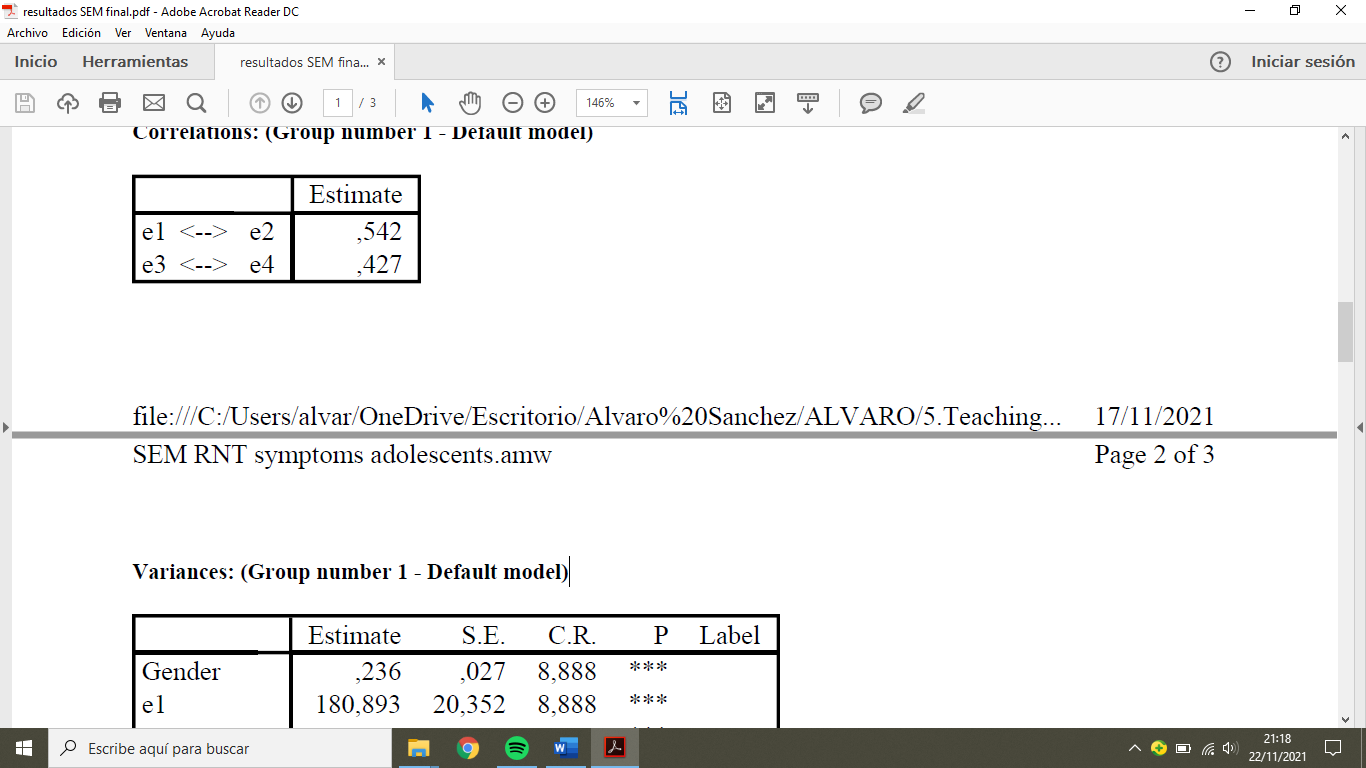


Variances: (Group number 1 - Default model)


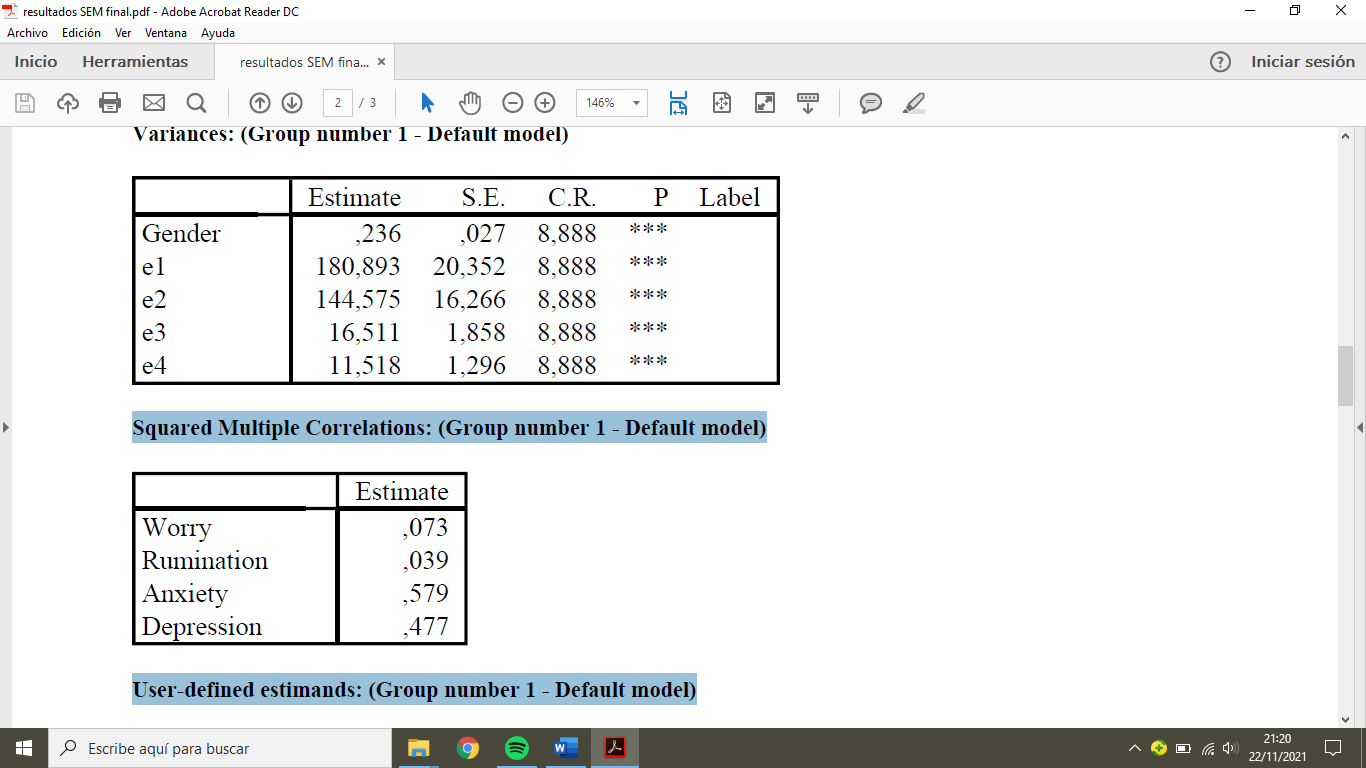


Squared Multiple Correlations: (Group number 1 - Default model)


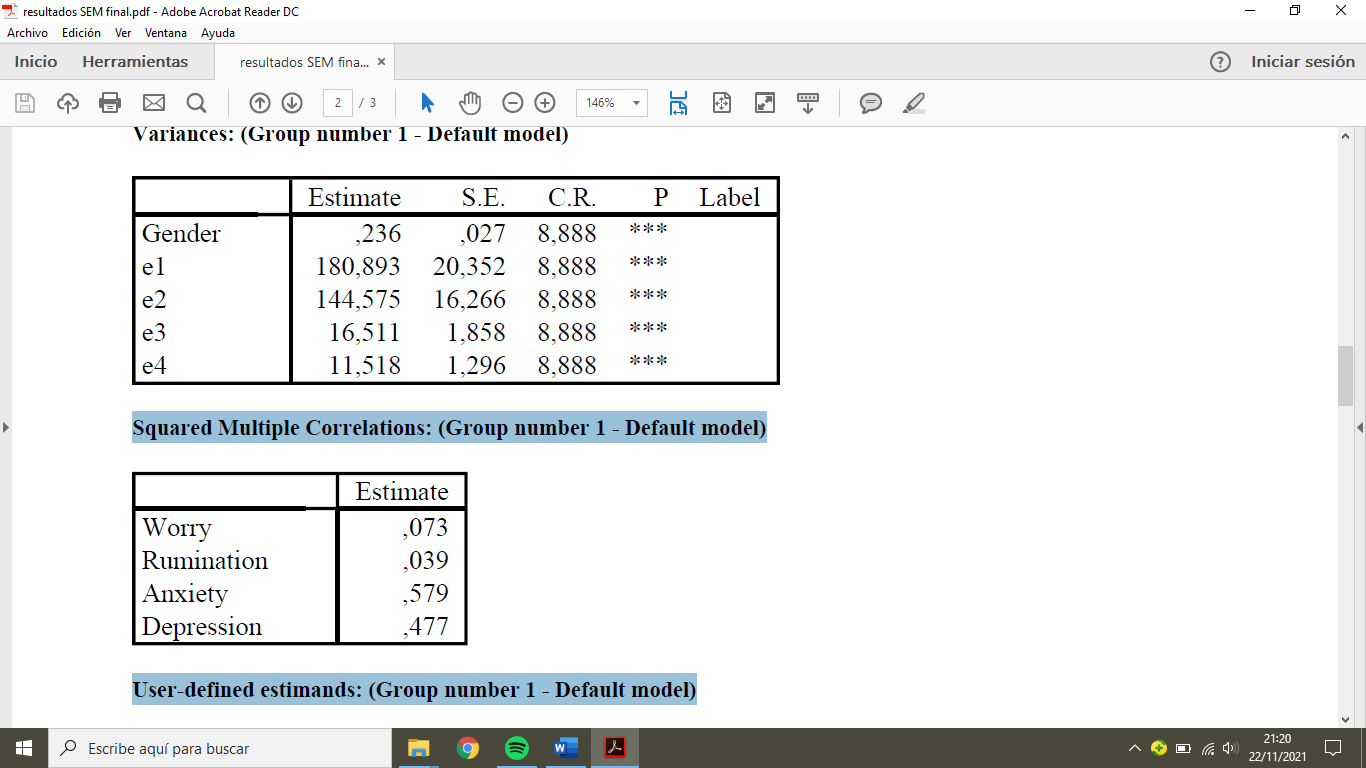


User-defined estimands: (Group number 1 - Default model)


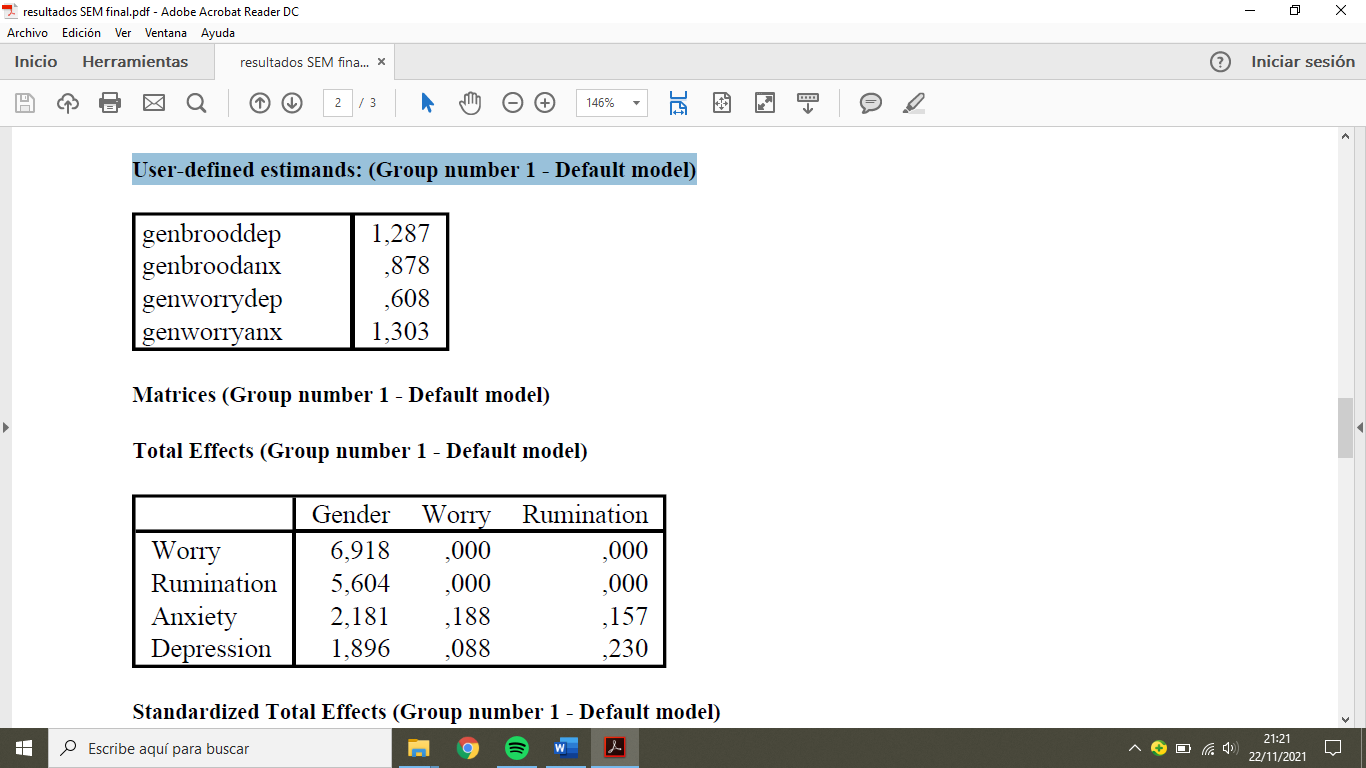


Matrices (Group number 1 - Default model)

Total Effects (Group number 1 - Default model)


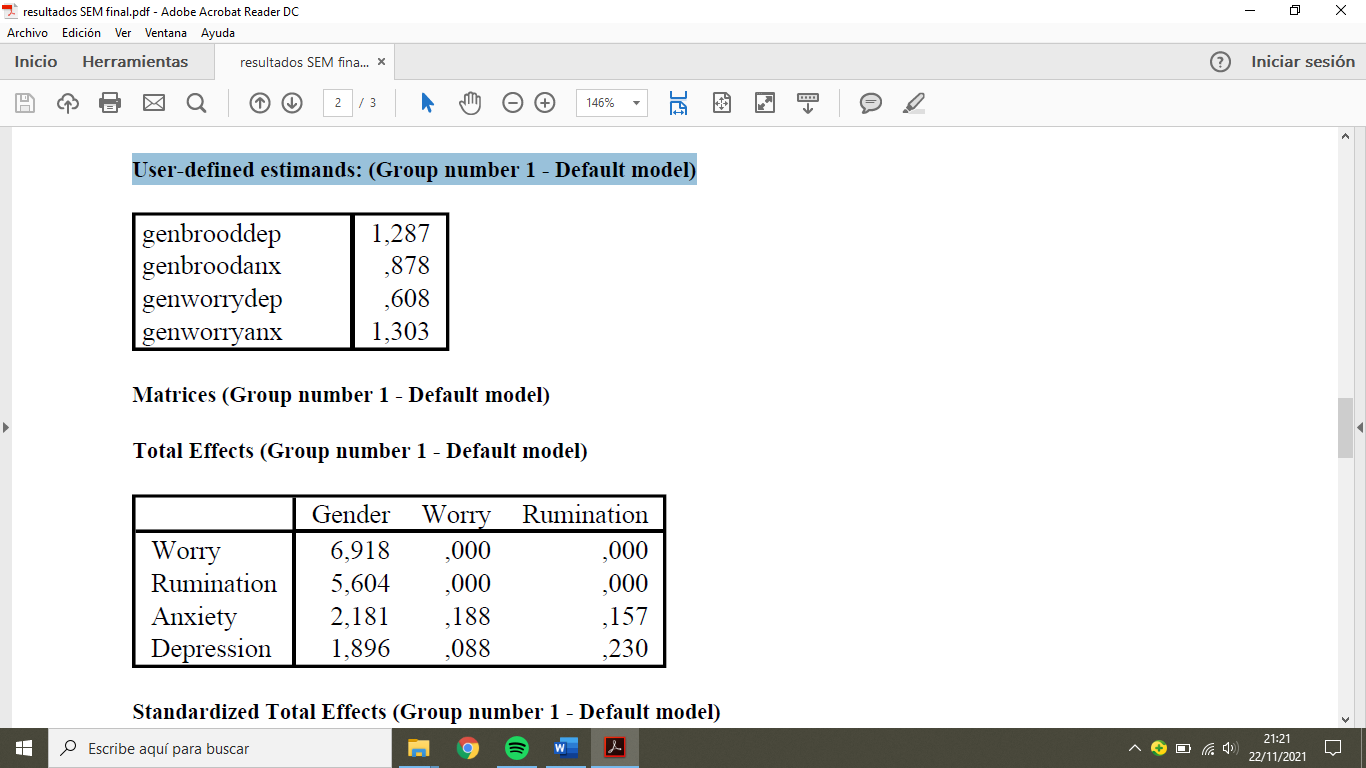


Standardized Total Effects (Group number 1 - Default model)


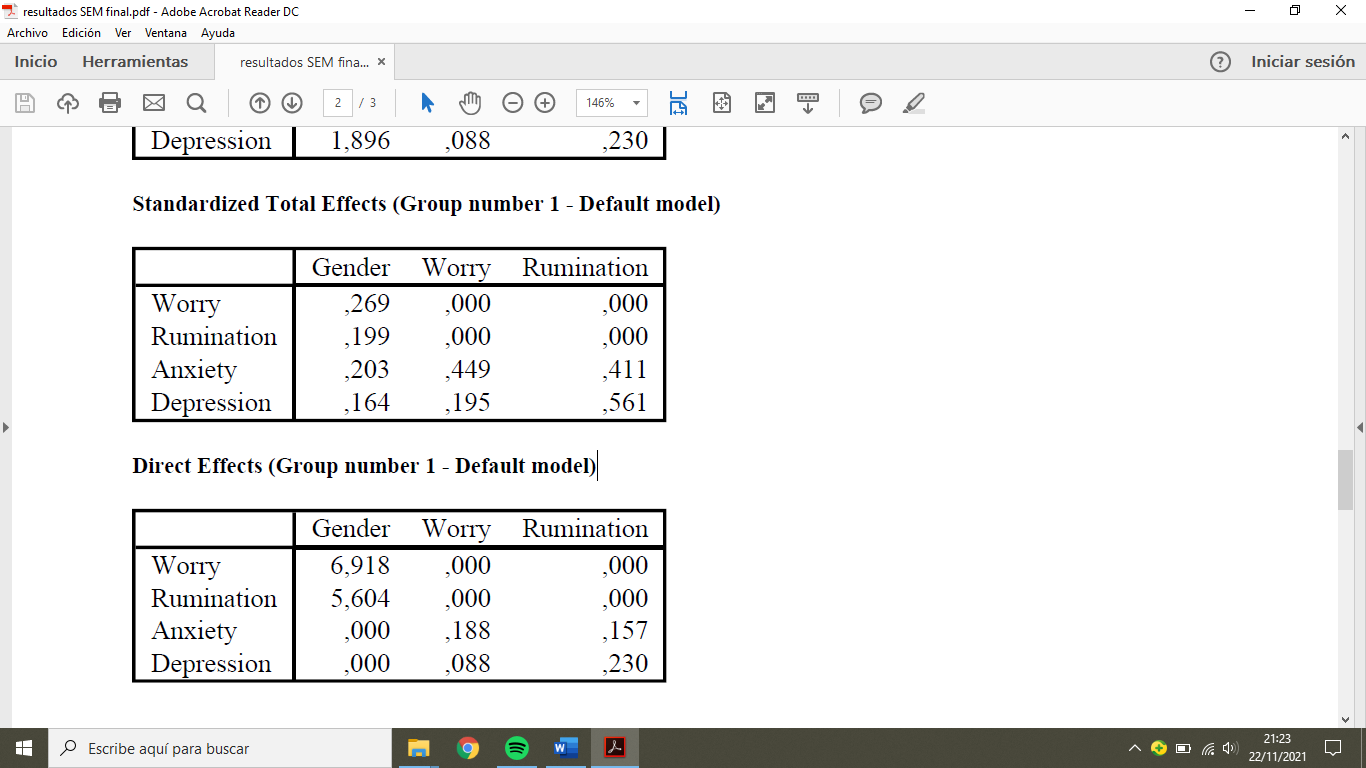


Direct Effects (Group number 1 - Default model)


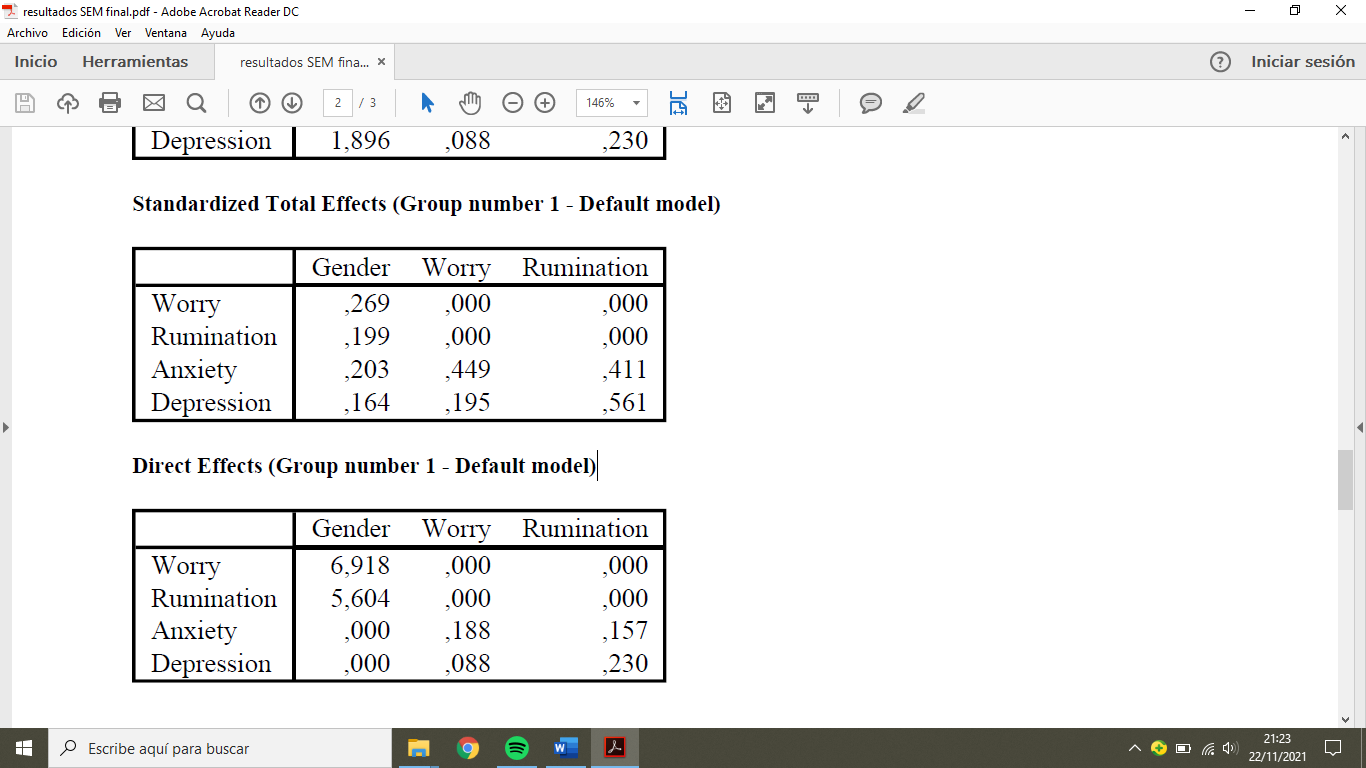


Standardized Direct Effects (Group number 1 - Default model)


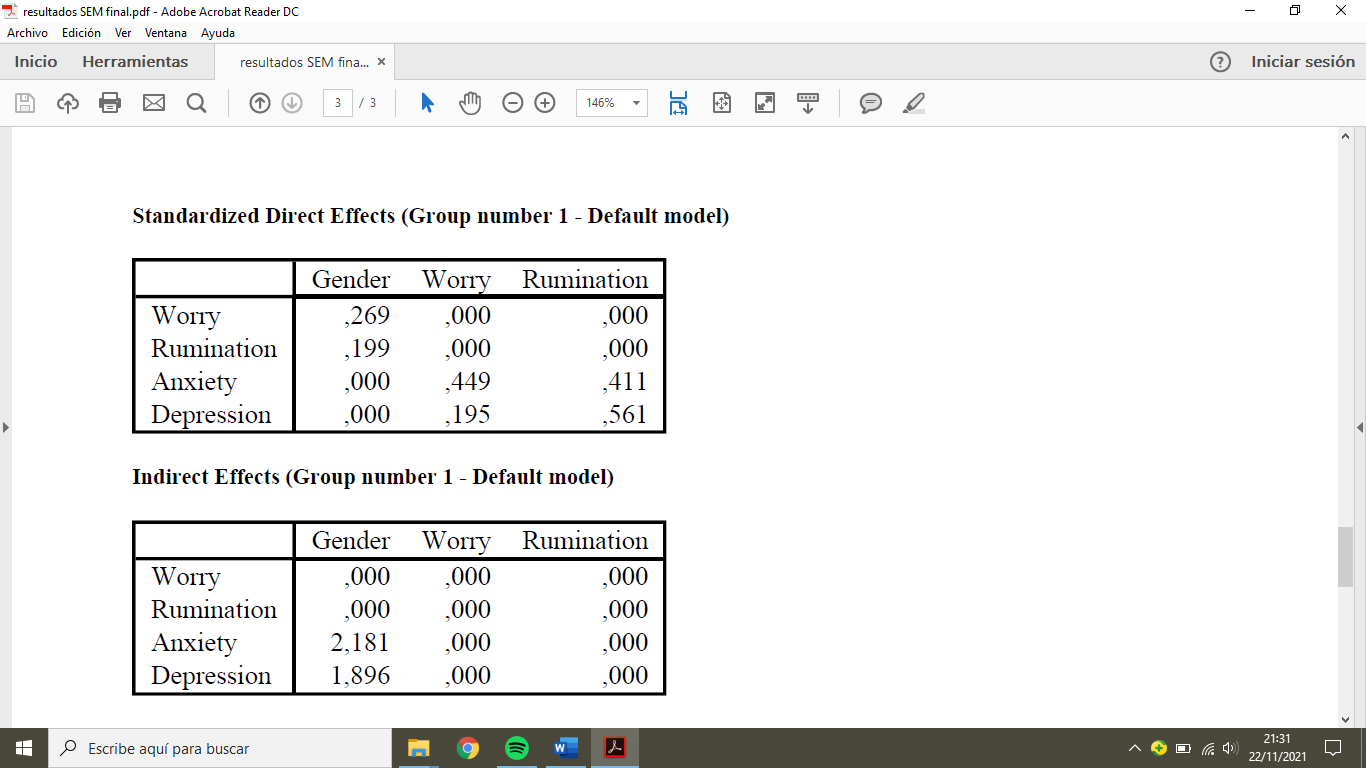


Indirect Effects (Group number 1 - Default model)


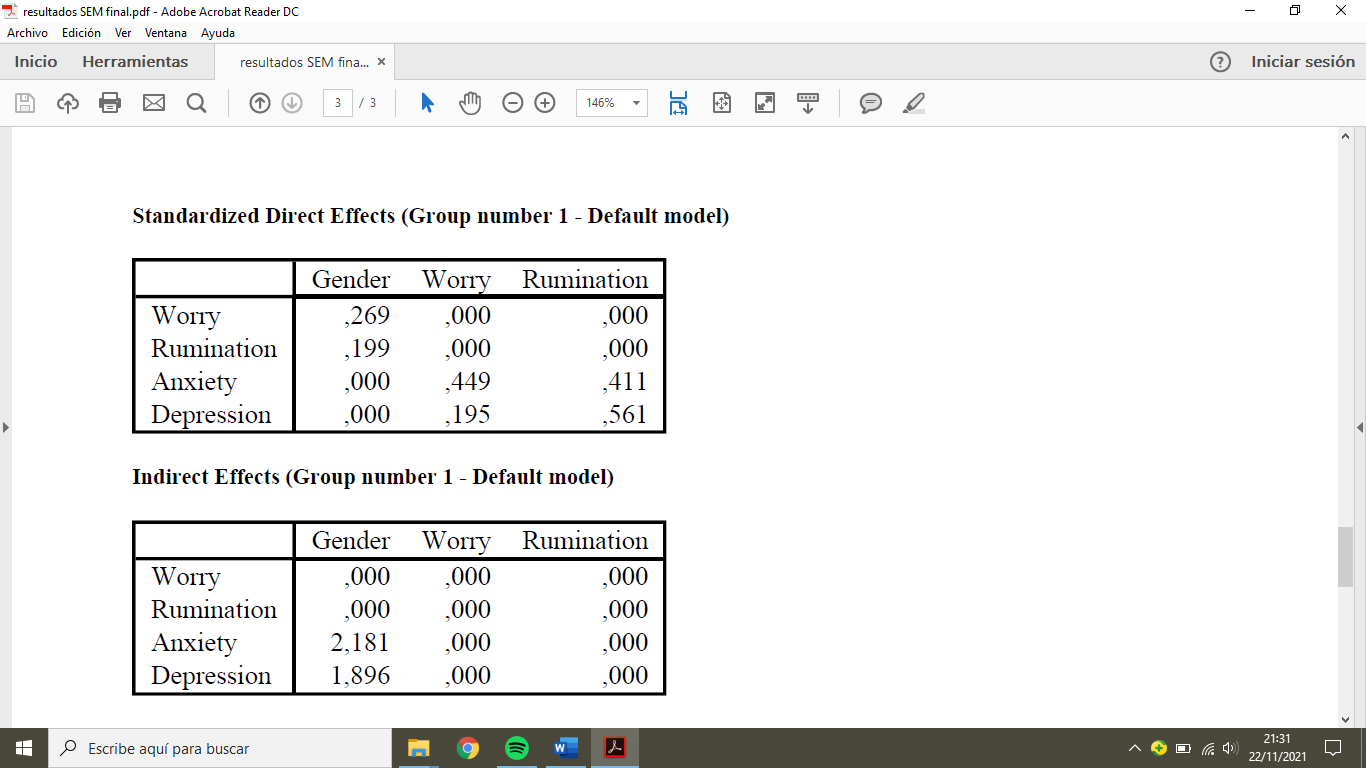


Standardized Indirect Effects (Group number 1 - Default model)


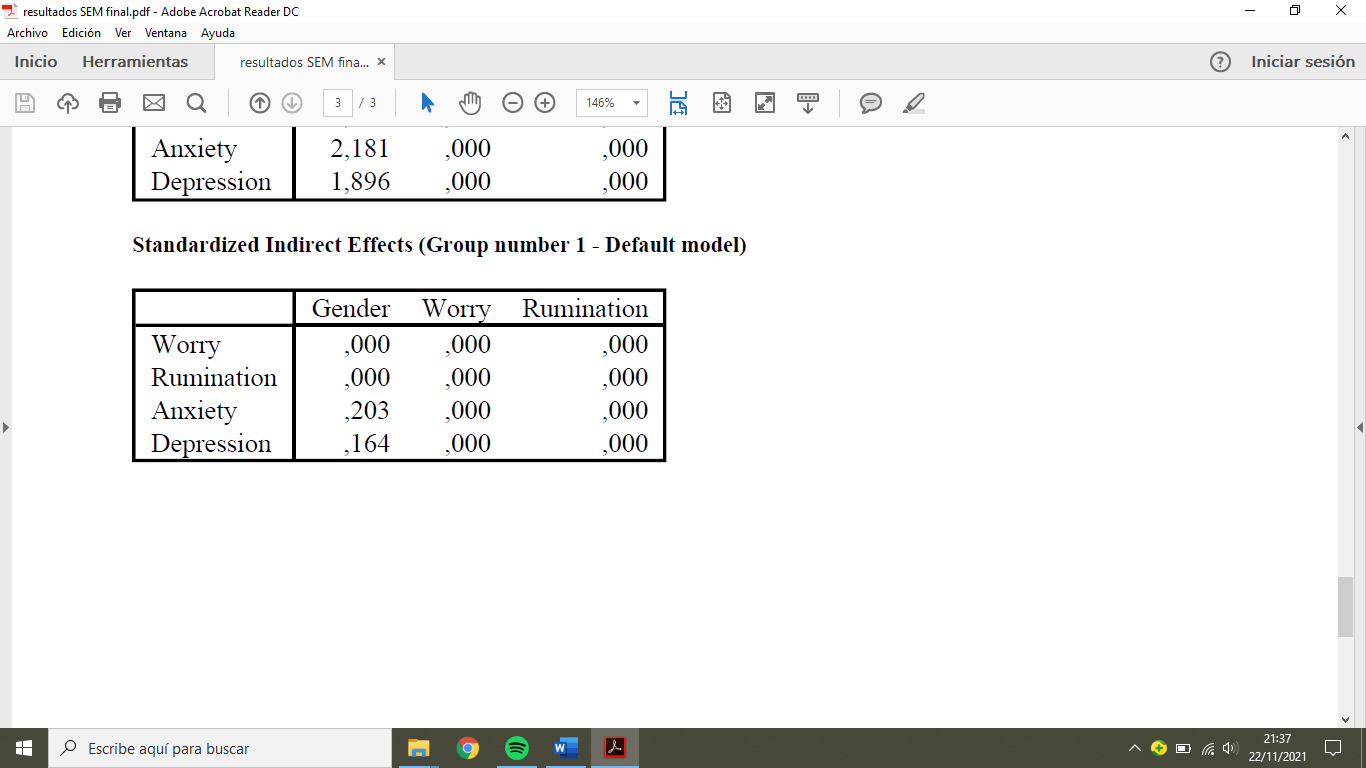

Supplement: Supplementary file 1 — Supplementary file1 (DOCX 1040 KB) [file 41811_2022_133_MOESM1_ESM.docx]
